# Mechanism of Soy Isoflavone Daidzein-Induced Female-Specific Anorectic Effect

**DOI:** 10.3390/metabo12030252

**Published:** 2022-03-16

**Authors:** Mina Fujitani, Takafumi Mizushige, Sudhashree Adhikari, Keshab Bhattarai, Taro Kishida

**Affiliations:** 1 Graduate School of Agriculture, Ehime University, 3-5-7 Tarumi, Matsuyama 790-8566, Japan; fujitani.mina.uu@ehime-u.ac.jp; 2 Department of Applied Biological Chemistry, School of Agriculture, Utsunomiya University, 350 Minemachi, Utsunomiya 321-8505, Japan; mizushige@cc.utsunomiya-u.ac.jp; 3 The United Graduate School of Agricultural Sciences, Ehime University, 3-5-7 Tarumi, Matsuyama 790-8566, Japan; sudhashreeadhikari@gmail.com (S.A.); keshab.bhattrai@gmail.com (K.B.)

**Keywords:** daidzein, equol, appetite, hypothalamus, gastric emptying

## Abstract

Epidemiological studies suggest that regular intake of soy isoflavone exerts a preventive effect on postmenopausal obesity and other forms of dysmetabolism. Estrogens inhibit eating behavior. Soy isoflavones may act as estrogen agonist in estrogen-depleted conditions, whereas they may either act as an estrogen antagonist or be ineffective in estrogen-repleted conditions. We investigated the effects of dietary soy isoflavone on food intake under various estrogen conditions using male, ovariectomized (OVX), and non-OVX female rats, and compared the effects with those of estradiol. We found that soy isoflavones reduced food intake in females specifically, regardless of whether ovariectomy had been performed, whereas subcutaneous implantation of estradiol pellet did not reduce food intake in intact female rats, but did so in OVX female and male rats. Contrary to this hypothesis, the reduction in food intake may not be caused by the estrogenic properties of soy isoflavones. It is of great interest to understand the mechanisms underlying the anorectic effects of soy isoflavones. In this non-systematic review, we summarize our recent studies that have investigated the bioactive substances of anorectic action, pharmacokinetic properties of soy isoflavones, and the modification of central and peripheral signals regulating appetite by soy isoflavones, and selected studies that were identified via database mining.

## 1. Introduction

Epidemiological and experimental researchers have provided extensive information on the beneficial effects of soy isoflavones on human health [1,2,3,4,5]. A possible explanation for some of these effects is the antiestrogenic property of soy isoflavones [3]. It is well-known that mammary tumor cells proliferate in response to estrogen, and soy isoflavones inhibit the estrogen-dependent proliferation in MCF-7 cells [6]. In contrast, preventive effect of soy isoflavones on osteoporosis in estrogen deplete condition in postmenopausal women and in ovariectomized (OVX) animals have been reported [7,8]. It is suggested that soy isoflavones act as estrogen antagonists in estrogen-dependent uterine contraction in intact rats [9]. Both genistein and daidzein, major soy isoflavones, have structural similarity to 17β-estradiol, and they have binding capacity to estrogen receptors and activate related gene expressions [1]. The binding affinities of genistein and daidzein are ≈1000-fold weaker than that of 17β-estradiol [10,11,12]. Therefore, in estrogen-depleted conditions, they may act as estrogen agonist, whereas they may either act as estrogen antagonist or be ineffective in estrogen-repleted conditions. In immature female mice, the uterine weight increased by dietary administration of soy, and more strongly increased by dietary administration of the synthetic estrogen diethylstilbestrol; however, the effect of diethylstilbestrol was weakened by concurrent administration of soy [13].

The food intake pattern fluctuates greatly depending on the estrus cycle; there are many reports about hypophagia around the ovulation period, the time of highest 17β-estradiol levels, in female rats and other animals, as well as in humans [14,15,16,17]. Decrease in food intake and consequent weight loss have been observed by many researchers when estrogen level is increased [14,15]. In female rats, subcutaneously administrated 17β-estradiol reduced food intake in the early stages [18], and in OVX rats, the restoration of estrous cycle by cyclic treatment of estradiol decreased food intake and body weight [19]. Decrease in food intake by estradiol administration was canceled in estrogen receptor-α knockout mice, suggesting that estrogen receptor-α mediated the decrease in food intake caused by estradiol administration [20].

Epidemiological studies suggest that regular dietary intake of soy isoflavone has a preventive effect on obesity and other dysmetabolism in postmenopausal women [21,22]. We examined whether soy isoflavones could affect food intake and body weight gain via their estrogenic properties. We found that the soy isoflavone daidzein, but not genistein, has a female-specific effect of reducing food intake in rats; however, contrary to the hypothesis, the reduction in food intake may not be caused by the estrogenic properties of soy isoflavone. This review aims to summarize our previous studies and selected studies identified via database mining and discusses the mechanisms by which soy isoflavone reduces food intake, specifically in female rats, but it does not aim to provide a systematic review.

## 2. The Anorectic Effect May Not Be Caused by Estrogenic Properties of Soy Isoflavones

**Figure 1:**
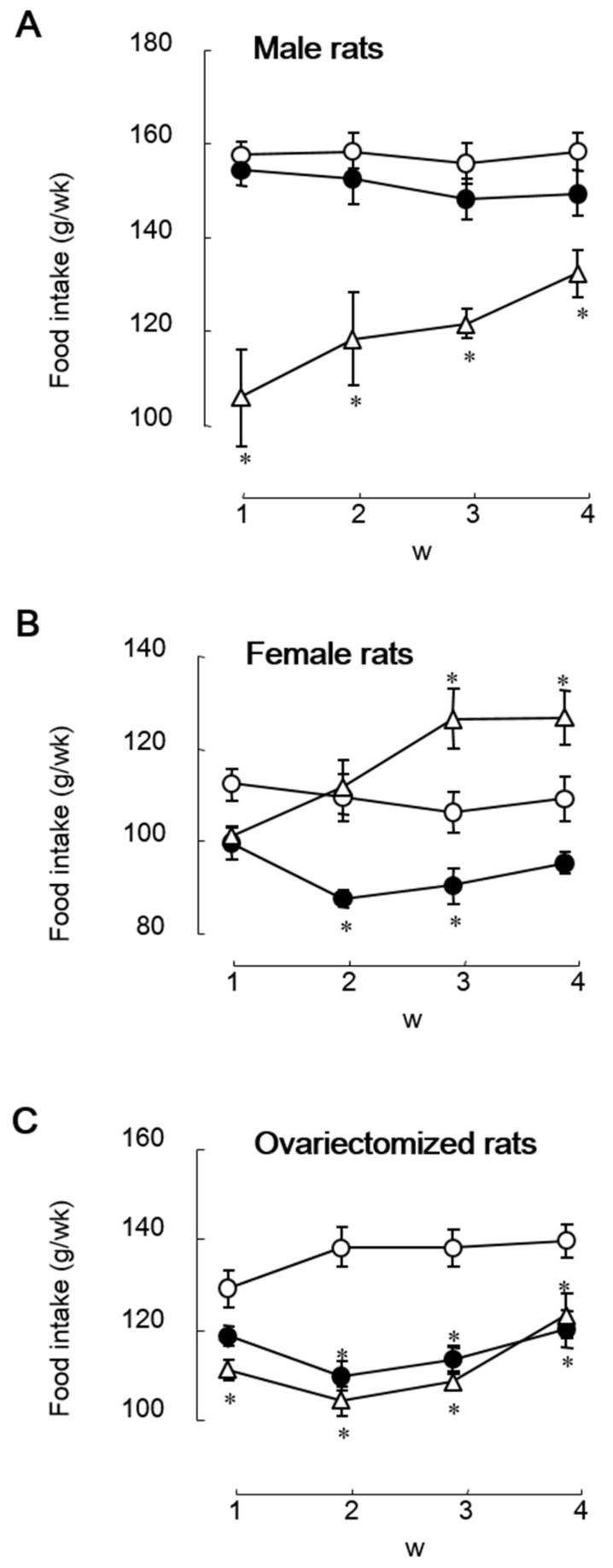
Weekly changes in food intake in male (**A**), female (**B**), and ovariectomized rats (**C**) fed on a diet containing isoflavone aglycone-rich fermented soybean extract (containing 300 mg isoflavones/kg diet, closed circle) or the control diet without (open circle) or with (open triangle) subcutaneous implantation of estradiol pellet (4.2 μg/rat/day) for 4 weeks [23]. Each value represents the mean ± standard error. Asterisks show significant difference relative to rats fed on the control diet without subcutaneous implantation of estradiol pellet, determined using three-way ANOVA with repeated measures, followed by Student’s *t*-test with Bonferroni corrections. *, *p* < 0.05.

We are interested in the estrogen-agonistic and -antagonistic actions of soy isoflavones regarding the roles in eating behavior. We investigated the effects of dietary soy isoflavone on food intake and body weight gain under various conditions of endogenous estrogen using female (standard estrogen condition), male (naturally low estrogen condition), and OVX (artificially induced estrogen-deficiency condition) rats and compared the effects and the estrogenic properties with those of 17β-estradiol. In early studies, we used isoflavone aglycone-rich fermented soybean extract (FSBE). In a 4 week feeding study, dietary FSBE (300 mg isoflavones/kg diet) significantly reduced food intake in female rats with and without OVX, but did not in male rats, whereas subcutaneous implantation of estradiol pellet (4.2 μg/rat/day) significantly reduced food intake in OVX female rats and male rats, but did not in intact female rats (Figure 1) [23]. It was contrary to our expectation that estrogen and isoflavones had the same effect on food intake. Moreover, dietary FSBE did not increase uterus weight in female rats, regardless of whether they were OVX or not, whereas subcutaneous implantation of estradiol pellet significantly increased the uterus weight only in OVX female rats [23]. Ovariectomy strongly decreases estrogen secretion and slightly increases estrogen sensitivity or estrogen receptor expression [24,25]. Ovariectomy-atrophied uterine weight should increase, as well as estrogen, if isoflavones reduce food intake through estrogenic effects. The anorectic effect of soy isoflavone may not be attributed to a purely estrogenic action.

Soy isoflavones have an unpleasant bitterness; therefore, the decrease in food intake could possibly be caused by the taste aversion. De Beun et al. [26] and Peeters et al. [27] previously reported a sex-dependent difference in taste aversion; therefore, it is also possible that an aversion to the bitter taste of soy isoflavones is exhibited specifically in females. To clarify this, 7-week-old male and female rats were intubated with carboxymethylcellulose suspension or suspension containing FSBE once a day for 4 weeks using a stomach tube so as to avoid the possible effect of taste aversion, resulting in intubated FSBE reducing food intake only in female rats, similar to that observed with its dietary intake [23]. The reduction in food intake in female rats may not be explained by the taste aversion to soy isoflavones.

We investigated daidzein and genistein, the potential effective components in FSBE, to verify if they are responsible for the anorectic effect. Daidzein (150 mg/kg diet), genistein (150 mg/kg diet), daidzein and genistein (1:1, 300 mg/kg diet), or control diets were fed to non-OVX and OVX rats for 4 weeks. Dietary daidzein, but not genistein, had an anorectic effect in OVX and non-OVX female rats in a similar feeding study to which we found the effect of dietary FSBE on food intake [28]. Intestinal bacteria convert daidzein to equol or to *O*-desmethylangolensin (*O*-DMA) [29,30,31,32]. Equol possesses antioxidant, anti-cancer, and anti-osteoporosis effects [33]. We searched a database of Scopus using the following search terms: (TITLE (equol) AND TITLE (woman OR female) AND TITLE-ABS-KEY (body-weight-gain OR fat-mass OR fat-accumulation)). We found seven publications. Of these, we excluded reviews. Equol administration decreased food intake [34], body weight gain [34,35,36], and fat accumulation [34,35,37] in female rats and mice. The protective effects of soy isoflavones on adiposity are suggested to depend on an individual’s equol-producing capacity in early postmenopausal women [38]. On the other hand, Liu et al. reported that six month consumption of whole soy and purified daidzein had no improvement on body weight and composition in equol-producing postmenopausal women [39]. Dietary FSBE or daidzein elevates the serum equol concentration to much higher levels than daidzein levels in male, female, and OVX rats. Although results from clinical trials are still controversial, results from in animal studies from our and other laboratories indicate that equol is possibly responsible for the anorectic effect.

## 3. Certain Amount of Equol Accumulation in Enterohepatic Circulation May Be Required to Exhibit the Anorectic Effect Caused by Dietary Daidzein

**Figure 2:**
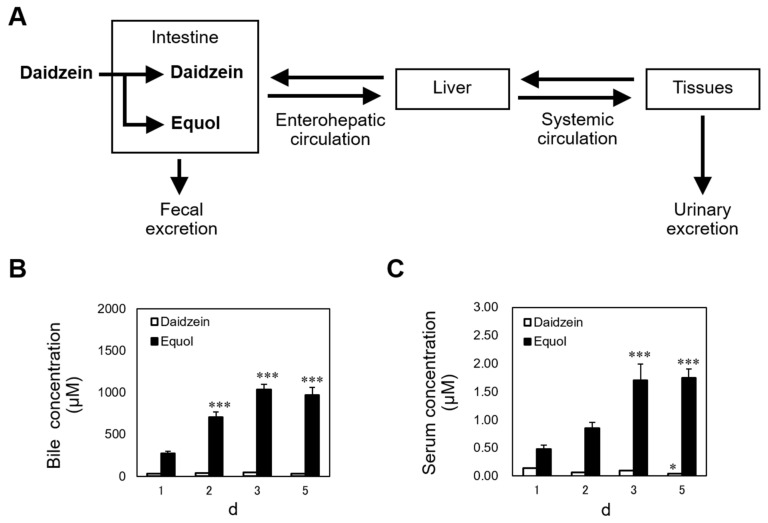
Diagram of the distribution of daidzein and equol in the body (**A**). Daily changes in daidzein and equol concentrations in bile (**B**) and serum (**C**) in intact female rats fed a diet containing 150 mg/kg daidzein [42]. Each value represents the mean ± standard error. Asterisks show significant difference relative to day 1, determined by Dunnett’s multiple comparison test. *, *p* < 0.05; ***, *p* < 0.001.

To understand the mechanism of the anorectic action of dietary daidzein, it is necessary to determine the distribution of daidzein and equol in the body. Although little is known about the distribution of equol in the body, the second and third peaks in plasma concentration-time profile of equol after oral administration indicates that it undergoes enterohepatic circulation (EHC) [40]. EHC is often associated with multiple peaks and a longer apparent half-life in a plasma concentration-time profile [41]. We fed sham-operated and OVX rats the daidzein (150 mg/kg diet) or control diets for 7 days, finding that dietary daidzein increased serum and bile concentrations of equol to far higher levels than those of daidzein itself, and the equol concentration was several hundred-fold higher in the bile than in the serum of both non-OVX and OVX rats, suggesting that a substantial proportion of dietary daidzein was converted to equol, which underwent efficient EHC, and only a small part of equol leaves EHC and enters the systemic circulation. EHC may influence drug concentrations in the body through delayed elimination [41]. We fed female rats the daidzein, equol (both 150 mg/kg diet), or control diet for 1, 2, 3, or 5 days. Continuous intake of equol resulted in daily increases in serum and bile concentrations for 5 days (Figure 2) [42]. The accumulation of equol in the body may be facilitated by efficient EHC. In addition, our results suggest that continuous daidzein intake for several days is required for its anorectic effect to occur [42]. This was also the case for dietary equol; continuous intake for 3 days was required [42]. It is possible that certain amount of equol accumulation in EHC may be required to exhibit the anorectic effect. Accumulation of equol in EHC may lead to an increase in its abundance in the small intestinal lumen. The small intestine releases hormones that regulate satiety. It is speculated that accumulation of equol in EHC induces its anorectic effect via increased release of satiety signals such as cholecystokinin (CCK), glucagon-like peptide 1 (GLP-1), and peptide YY (PYY).

## 4. Daidzein Alters Gene Expression of Hypothalamic Appetite-Related Neuropeptides

Appetite is regulated by a complex system of central and peripheral signals that interact to modulate the individual response to nutrient ingestion. Peripheral regulation includes satiety signals (CCK, GLP-1, PYY) and adiposity signals (leptin and insulin), while central control is accomplished by several effectors, including the neuropeptidergic, monoaminergic, and endocannabinoid systems [43]. Firstly, we investigated whether CCK and leptin signaling contribute to the anorectic effect of dietary daidzein in a feeding study (150 mg/kg diet) for 5 weeks using spontaneous hormone receptor deficient rats, the Otsuka Long–Evans Tokushima Fatty rats (CCK type 1 receptor (CCK1R)-deficient), and the obese Zucker fa/fa rats (the long form of the leptin receptor (ObRb)-deficient). Dietary daidzein reduced food intake with or without CCK1R [44]. Dietary daidzein significantly reduced food intake in OVX leptin-ObRb signaling-deficient rats [44]. These results significantly indicate that these two major appetite-regulators do not necessarily contribute to the mechanism anorectic effect of dietary daidzein. Dietary daidzein did not reduce food intake and body weight gain in sham-operated Zucker *fa/fa* rats in spite of the fact that dietary daidzein significantly reduced these parameter in OVX Zucker fa/fa rats [43]. We speculate that the absence of an anorectic effect might be associated with the serum equol concentration. Generally, rats are considered to be highly equol-producing animals. However, we showed that serum equol level in Zucker strain rats was lower than those in the Long–Evans strain rats and Sprague–Dawley (SD) rats [23,42,44]. Our results suggested that the disappearance of the effect of the reducing effect on food intake in sham-operated Zucker fa/fa rats was due to the lower serum equol concentration than that in sham rats.

**Figure 3:**
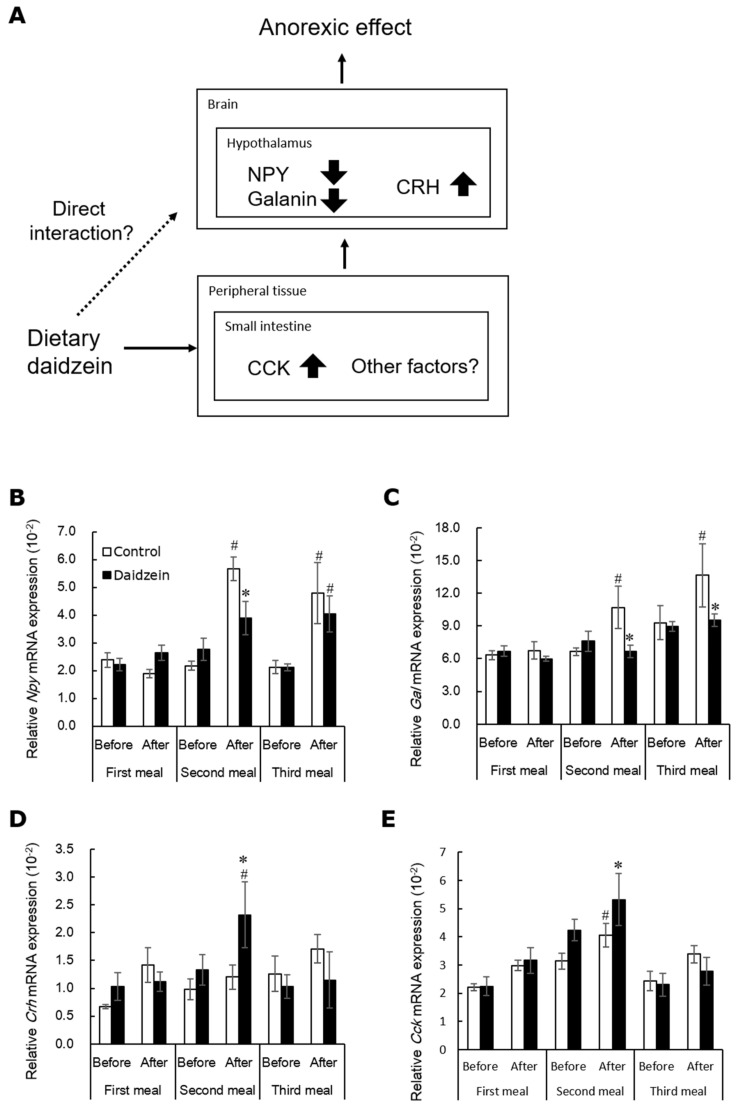
Diagram of a hypothesis regarding the mechanisms of the anorectic effect of daidzein (**A**). Changes in expression of neuropeptide-Y (*Npy*, (**B**)), galanin (*Gal*, (**C**)), and corticotrophin releasing hormone (*Crh*, (**D**)) mRNA in the rat hypothalamus, and cholecystokinin (*Cck*, (**E**)) mRNA in the rat upper small intestine before and after each meal [48]. Each value represents the mean ± standard error. Asterisks show a significant difference compared to the corresponding control group, and number signs show a significant difference compared to the corresponding before-meal group, determined by three-way ANOVA with Bonferroni corrections. * and #, *p* < 0.05.

Next, we determined whether the decreasing effect of dietary daidzein on food intake is mediated by the gene expression of appetite-related neurotransmitters adopting the daily three meals feeding. This feeding method enables the evaluation of changes of gene expression appetite-related neurotransmitters just before and after ingestion of the test meal [45,46,47]. We fed female rats the daidzein (150 mg/kg diet) or control diet on the daily three meals feeding for 13 days. Dietary daidzein-induced anorectic effect was observed mainly during the second meal, but rarely during the first and third meals [48]. Interestingly, our findings suggested that daidzein attenuates the postprandial increase in mRNA expression of *Npy* and *Gal*, and increased *Crh* mRNA expression in the hypothalamus (Figure 3B–D), concomitantly with the reduction in food intake [48]. NPY induces potent hyperphagia and obesity [49,50]. In the hypothalamus, galanin has been reported to interact with NPY. It has been immunohistochemically demonstrated that NPY and galanin colocalize in hypothalamic synapses and that NPY-containing axons proximate closely to galanin-containing somas and dendrites [51,52]. NPY administration stimulates galanin secretion in galanin neurons [52]. It has been reported that NPY and galanin collaborate to stimulate secretion of luteinizing hormone [53]. Our results suggested that *Gal* mRNA expression is caused by dietary daidzein-induced increase in *Npy* mRNA expression. Dietary daidzein significantly increased *Cck* mRNA expression in the upper small intestine before and after the second meal (Figure 3E) [48]. However, as described above, we have demonstrated that CCK signaling is not essential for the anorectic effects of daidzein [44]. Overall, we speculated that dietary daidzein-induced alterations in the hypothalamic dynamics of *Npy Gal*, and *Crh* mRNA expression may play certain role in the decreasing effect of daidzein on food intake.

Regarding direct effect of equol on brain system, we searched a database of Scopus using the following search terms: (TITLE (equol) AND TITLE-ABS-KEY (neuron AND in AND vitro)). We found six articles and narrowed them down these to five articles that examined the direct effect of equol in neurons and glial cells. In vitro studies demonstrated that the addition of free equol to culture media protected neurons against neuroinflammatory injury mediated by LPS-activated microglia [54], β-amyloid-induced cytotoxicity and cell-cycle reentry [55], hypoxia/reoxygenation injury [56], and HIV-1 Tat and cocaine induced synaptopathy [57]. Furthermore, the addition of a mixture of genistein, daidzein, and equol increased mitochondrial respiration in cultured primary neurons [58]. Although free equol may have a better blood–brain barrier permeability than other phytoestrogen [59], equol glucuronide conjugates are the predominant existent form in rat plasma after oral administration [40]. It has generally been assumed that the physicochemical properties of glucuronides are incompatible with passage though the BBB [60,61]. Previous studies have suggested that the limited accumulation of genistein in brain tissue may reflect poor penetration of isoflavones into the central nervous system of adult rats [62]. Although there is no report about equol levels in brain tissue, it is speculated that the direct effect of equol on brain system may be limited. Therefore, we infer that equol may alter the hypothalamic dynamics of *Npy*, *Gal*, and *Crh* mRNA expression by indirect effects on brain function.

## 5. Daidzein Induces the Anorectic Effect by Delaying Gastric Emptying

**Figure 4:**
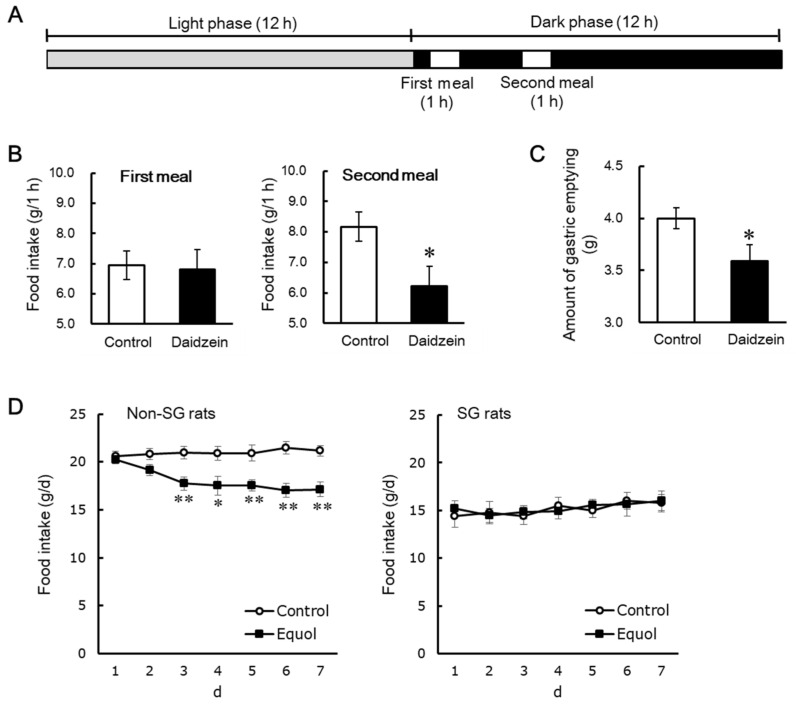
Diagram for feeding schedules (**A**). Food intake during the first and second meals (**B**), and the amount of gastric emptying (**C**) in daily two meal-fed ovariectomized rats. Daily changes in food intake in ovariectomized rats with and without sleeve gastrectomy (**D**) [67]. Each value represents the mean ± standard error. Asterisks show significant difference relative to the control group, determined by an unpaired Student’s *t*-test. *, *p* < 0.05; **, *p* < 0.01. non-SG, intact groups; SG, sleeve gastrectomy-operated groups.

As described above, dietary daidzein decreased food intake during the second meal, but not during the first meal in daily three meals feeding in rats [48]. It might be explained that dietary daidzein may decrease food intake during the time when gastric contents remain to some extent. Clinical and basic studies suggest that delayed gastric emptying may accelerate satiety and decrease food intake [63,64,65,66]. We evaluated the contribution of gastric emptying to decreasing effect of dietary daidzein on food intake in daily two meal feeding in rats, applying a simpler system than three meal feeding. OVX rats were fed the daidzein (300 mg/kg diet) or control diets on the daily twice meal feeding for 5 days, resulting in dietary daidzein significantly decreasing food intake during the second meal after the three days of the experimental period, but not doing so during the first meal at any time in the experimental period (Figure 4B) [67]. In fifth day of the experimental period, the amount of gastric emptying 2 h after the end of the first meal significantly reduced by dietary daidzein (Figure 4C) [67]. Dietary daidzein might decrease food intake only in the presence of residual gastric contents; furthermore, delayed gastric emptying may attribute to the decreasing effect of daidzein on food intake. To address this hypothesis, we used sleeve gastrectomy (SG) rats with resected 50% of the total stomach including gastric fundus and part of gastric body avoiding to accumulate gastric contents. We used equol instead of daidzein, as SG may suppress the intestinal bacterial conversion of daidzein to equol. Non-SG and SG rats were fed the equol (150 mg/kg diet) or control diets for 7 days, resulting in dietary equol significantly reducing daily food intake in non-SG rats but not in SG rats (Figure 4D) [67]. The capacity to accumulate food in the stomach may be required for the anorectic effect of equol to occur. These results support the hypothesis that the anorectic effect of dietary daidzein is attributed to delayed gastric emptying. Although the mechanisms involved are unclear, we demonstrated that dietary daidzein decreased *Npy* mRNA and increased *Crh* mRNA in the hypothalamus, as described above [48]. Nakade et al. reported that central glucagon-like peptide-1 delays solid gastric emptying via central CRH [68]. Central NPY counteracts the biological actions of CRH [69], and NPY release, acting through the Y1 receptor, inhibits gastric distension-induced pyloric relaxation in rats exposed to acute elevations in blood glucose concentrations [70].

## 6. Conclusions and Future Prospectus

We found that dietary soy isoflavone and its daily intubation reduced food intake in female rats with and without ovariectomy, but not in male animals [23]. However, continuous administration of estradiol reduced food intake in male rats and in OVX rats, probably because of low endogenous estrogen levels [23]. It is possible that the reduction in food intake in soy isoflavone-fed female rats was not due to a purely estrogenic function.

We found that dietary daidzein reduced food intake in female rats [28,42,44,48,67], but not genistein [28]. Dietary soy isoflavone increases the serum concentration of equol to far higher levels than that of daidzein in male, female, and OVX rats [23]. Dietary equol reduced food intake in OVX rats [34,67]. These results indicated that equol is responsible for the anorectic effect. The anorectic effects of soy isoflavone may largely depend on its metabolism by intestinal bacteria, as well as pharmacokinetic properties, especially absorption and distribution to the target tissue. It is suggested that a substantial proportion of dietary daidzein was converted to equol, and that continuous intake of daidzein induced accumulation of equol in EHC to far higher levels than that of daidzein itself, and only a small portion of equol escaped EHC and reached systemic circulation in female rats [42]. Equol primarily occurs in the form of major metabolites such as glucuronides and sulfates in rats and humans [40,71,72]. The biological activity of these equol conjugates is of interest. The differences in disposition and biotransformation of daidzein depending on gender were reported by Bayer et al.; they found that daidzein, daidzein-glucuronide, and daidzein-sulfate were excreted in the urine of male rats fed on a diet containing daidzein, whereas only unmetabolized daidzein and daidzein-glucuronide were excreted in the urine of female rats [31]. It is possible that there are differences in the disposition and biotransformation of equol depending on gender. Further studies are needed to determine gender differences in the metabolic profile of equol.

We found that daidzein-induced changes in the dynamics of *Npy*, *Gal*, and *Crh* mRNA expression in the hypothalamus, an important appetite control center that integrates peripheral hormone signals and interacts with other brain regions, and *Cck* mRNA expression in the upper intestine using the three-meals-per-day feeding method [48]. In addition, our results suggested that dietary daidzein may induce the anorectic effect by delaying gastric emptying [67]. A high concentration of equol is poured into the upper gastrointestinal tract via bile in rats fed a diet containing daidzein. The upper intestinal tract plays an important role in sensing the arrival, amount, and chemical composition of a meal. Ingestion of a meal triggers a number of signals in the gastrointestinal tract. These signals are then transmitted to the brain where they contribute to food intake regulation by modulating appetite as well as feedback control of gastrointestinal functions [73]. During digestion, many hormones, including CCK and GLP-1, inhibit gastric emptying via the gastric inhibitory vagal motor circuit, and in the interdigestive period, the hormones ghrelin and motilin hasten gastric emptying by stimulating the gastric excitatory vagal motor circuit. The gastric inhibitory and excitatory vagal motor circuits are also connected to anorexigenic and orexigenic neural pathways, respectively [74]. Although we searched a database of Scopus using the following search terms: (TITLE (equol) AND TITLE-ABS-KEY (vagus-nerve)), there is no evidence of equol and/or daidzein binding site on the terminal of vagus neurons. However, our previous study demonstrated that a high amount of equol conjugates are absorbed from the intestine into EHC [42]. Vagal chemoreceptive fields are distributed in the mucosal lamina propria of the gut wall [75]. Equol conjugates may exist in high concentrations at the terminal of vagus neurons. Therefore, we infer that equol may control afferent activity of vagus nerve by direct effects or stimulating releases of gastrointestinal hormones. Further studies are necessary to determine whether the delaying effect of dietary daidzein on gastric emptying is mediated by stimulation of the gastric inhibitory vagal motor circuit or by inhibition of the gastric excitatory vagal motor circuit, which are connected with satiety- and hunger-associated neural pathways.

Equol [67] and estradiol [76,77] reduce food intake and delay gastric emptying. Equol [48] and estradiol [78] affect *Npy* and *Crh* mRNA expressions in the hypothalamus. However, they exert these effects presumably by different mechanisms. It has previously been shown that estradiol treatment increased *Pomc* mRNA expression in the hypothalamus in OVX mice [78], and decreased *Pmch* (pro-melanin-concentrating hormone) mRNA expression in the zona incerta (an area in the dorsal hypothalamus) in OVX rats [79]. Our results indicated that dietary daidzein did not significantly change *Pomc* and *Pmch* mRNA expression in the hypothalamus [48]. Estrogen acts via the estrogen receptors in the hypothalamus to reduce food intake [80]. On the other hand, the direct effect of equol on brain system may be limited as described above. We infer that equol may alter the hypothalamic appetite-related neuropeptides expressions via peripheral actions. However, contrary to this hypothesis, Nishimura et al. reported that estradiol reduced food intake and increased c-Fos expression in the suprachiasmatic nucleus, the center for circadian rhythm regulation, in OVX rats, and equol exerted similar effects in the vehicle-treated OVX rats but not in the estradiol-replaced OVX rats during the light phase, suggesting that equol exerts estradiol-like anorectic effect by modifying the diurnal feeding pattern [81].

To summarize the available evidence from human studies, we searched Scopus using the following search terms: (TITLE (soy OR isoflavone) AND TITLE (woman) AND TITLE (body-weight OR body-composition OR appetite OR food-intake OR energy-intake) AND TITLE-ABS-KEY (isoflavone)). We found 16 publications. Of these, we excluded reviews, notes, conference papers, and irrelevant articles. Epidemiological and experimental studies have examined the anti-obesity effects of soy isoflavones, but the results are controversial. Some studies suggest that dietary soy isoflavones may decrease body weight and abdominal adipose tissue gain in postmenopausal women [22,82], while others did not [39,83,84]. Regarding the pharmacokinetics of equol, we searched Scopus using the following search terms: (TITLE-ABS-KEY (equol AND pharmacokinetics) AND TITLE (woman)), found five articles, and narrowed them down to two pharmacokinetic studies of equol at oral doses. The systemic bioavailability of equol may be greater than daidzein and genistein [85,86], and equol may undergo EHC in postmenopausal women [85], in accordance with our results in rats [42]. We searched Scopus using the following search terms: (TITLE-ABS-KEY (isoflavone OR daidzein OR equol) AND TITLE-ABS-KEY (woman AND gastric AND emptying)). However, there was no report found regarding an observation in human subjects that dietary daidzein/equol can delay gastric emptying and affect hypothalamic control of appetite. It is, however, reported that soy isoflavones may increase preprandial PYY concentration [84] and decrease fasting ghrelin concentration [87] in postmenopausal women. Although results obtained in animal studies do not always accurately reflect outcomes in humans, it is likely that rodent models are the tool of choice for basic research into pharmacokinetics and feeding behavior. Table 1 summarizes the suggested mechanisms of daidzein/equol-induced anorectic effect. The information obtained from studies to determine the mechanisms of daidzein/equol-induced anorectic effect may be useful in the development of new approaches to induce appetite suppression and achieve satisfactory weight loss.

## Figures and Tables

**Table 1:** Suggested mechanisms of equol-induced anorectic effect.

Suggested Mechanisms	References
The accumulation of equol in enterohepatic circulation is needed to exert the anorectic effect caused by dietary daidzein.	[42,85,86]
Equol alters expression of hypothalamic appetite-related factors.	[48,81]
Equol induces the anorectic effect by delaying gastric emptying in OVX rats.	[67]
